# Differentiation-inducing and anti-proliferative activities of lupeol on canine melanoma cells

**DOI:** 10.1186/2193-1801-3-632

**Published:** 2014-10-25

**Authors:** Kikumi Ogihara, Yuko Naya, Yoshiharu Okamoto, Keishi Hata

**Affiliations:** School of Life and Environmental Science, Azabu University, 1-17-71 Fuchinobe, Chuo-ku, Sagamihara, Kanagawa, 252-5201 Japan; Department of Veterinary Clinical Medicine, School of Veterinary Medicine, Faculty of Agriculture, Tottori University, 4-101, Koyama-Minami, Tottori, 680-8553 Japan; Akita Research Institute of Food and Brewing, 4-26 Sanuki, Araya-machi, Akita, 010-1623 Japan

**Keywords:** Lupeol, Canine melanoma, Differentiation-inducing activity, Cytostatic effect, Nonsurgical treatment

## Abstract

Canine melanoma is the most common oral malignant tumor reported in the field of veterinary medicine. We found that lupeol, a lupine triterpene, inhibited mouse melanoma cell growth *in vitro* and *in vivo* by inducing cell differentiation. In the present study, we examined the differentiation-inducing activities of lupeol on 4 canine melanoma cells *in vitro* and *in vivo*.

The induction of canine melanoma cell differentiation by lupeol was confirmed by evaluating some differentiation markers such as tyrosinase with real-time RT-PCR. Furthermore, we transplanted canine melanoma cells into a severe combined immunodeficiency mouse, and studied the anti-progressive effects of lupeol on tumor tissue.

The gene expression of microphthalmia-associated transcription factor, tyrosinase, and tyrosinase-related protein-2, which are markers of pigment cell differentiation, was induced in 4 canine oral malignant melanoma cells by lupeol, and the agent markedly inhibited tumor progression in canine melanoma-bearing mice.

## Introduction

Melanoma is the most aggressive form of skin cancer that is derived from activated or genetically altered epidermal melanocytes (Oliveria et al. 2001). Human malignant melanoma is a highly metastatic cancer that is markedly resistant to chemotherapy with dacarbazine or temozolomide. Canine melanoma is the most common oral malignant tumor reported in the field of veterinary medicine (Bergman, 2007). It was reported that treatment of carboplastin significantly affects dogs with malignant melanoma, and achieved a response rate at 28% (Rassnick et al. 2001).

Lupeol, a plant pentacyclic triterpene, was previously shown to have various biological activities, such as anti-inflammatory (Fernández et al. 2001a,[b]), anti-cancer (Saleem 2009), and anti-metabolic syndrome (Sasaki et al. 2008; Hata et al. 2008; Itoh et al. 2009) activities. We previously demonstrated that lupeol inhibited mouse melanoma cell growth by inducing melanogenesis and suppressing the motility of cells (Hata et al. 2006). These findings revealed that lupeol induced the differentiation of melanoma cells into mature melanocyte-like cells *in vitro*. In our recent study, we showed that lupeol suppressed tumor growth in melanoma-bearing mice by attenuating proliferating cell nuclear factor (PCNA) and Ki67, which are highly expressed in melanoma or other tumors (Nitta et al. 2013). However, the effects of lupeol on other melanoma such as canine melanoma remain unclear. In the present study, we examined the differentiation-inducing activities of lupeol on canine melanoma cells *in vitro* and its anti-melanoma effects *in vivo*, prior to clinical trials on canine melanoma.

## Material and methods

### Cell culture

With prior written informed consents of animal owners, 4 melanoma samples were separately harvested after surgical procedures, and 4 canine oral malignant melanoma (cMEL) cell lines were established from these tumor samples. The three melanoma cell lines (cMEL-1, -3, and -4) were maintained in Dulbecco’s modified Eagle’s medium, and cMEL-2 cells were maintained in RPMI-1640 medium, respectively, supplemented with 10% fetal calf serum, 100 μg/ml of streptomycin, and 100 U of penicillin.

### Effect of lupeol on canine melanoma cell growth

Aliquots of 1 ml of cMEL-1-4 cells (1 × 10^4^ cells) were incubated with various concentrations of lupeol (Sigma-Aldrich, INC.) for 4 days, and the number of viable cells was counted by trypan blue exclusion method. IC_50_ values, representing the concentration that inhibited melanoma cell growth by 50%, were measured.

### RNA extraction and cDNA synthesis

Canine melanoma cells (2 × 10^4^ cells) in 10 cm-dishes (10 ml) were incubated with or without lupeol for 2 days. Total RNA was isolated using the QuickGene RNA cultured cell kit S (FUJIFILM, Co.). Template cDNA synthesis was performed with 5 μg of total RNA using the PrimeScript RT Reagent Kit (TAKARA BIO, INC.)

### Real-time RT-PCR

In a fluorescent temperature cycler (Chromo4; Bio-Rad Laboratories, Inc.), 2.5% of each RT reaction solution was amplified in 25 μl of 1 × SYBR Premix Ex Taq (TAKARA BIO, INC.) containing 0.2 μM of each primer. Samples were incubated in the thermal cycler for an initial denaturation at 95°C for 10 s, followed by 40 PCR cycles. Each cycle consisted of 95°C for 5 s and 60°C for 30 s. The oligonucleotide primers used in the present study are indicated in Table 1. To confirm the amplification of specific transcripts, melting curve profiles (cooling the sample to 60°C and heating slowly to 95°C with the continuous measurement of fluorescence) were produced at the end of each PCR. The relative expression level of each mRNA was normalized by the amount of GAPDH mRNA.

**Table 1:** **Oligonucleotide primers used in real-time RT-PCR**

Target gene	Primer sequence (5'-3')
GAPDH	S:-GCCAAGAGGGTCATCATCTC
	A:-GGCCCGTCCACGGTCTTCT
MITF	S:-GGGATTGATGGATCCTGCTTTG
	A:-GGCTGGACAGGAGTTGCTGA
TYR	S:-TTGGCAGATTGTCTGTAGCC
	A:-AGGCATTGTGCATGCTGCTT
TRP-2	S:-ACACAACACTGGCTGGGCCT
	A:-GAGATCTCTTTCCAGACACAAC
PCNA	S:-AGTCACATCGGAGATGCTGTTGTA
	A:-AGCTGAACTGGCTCATTCATCTCTA
Ki67	S:-AATCTCTGCTTCGGGTCTCCA
	A:-ACTCCGGTTTCAGACGACCAC

GAPDH Glyceraldehyde-3-phosphate dehydrogenase, MITF microphthalmia- associated transcription factor, TYR tyrosinase, TRP-2 tyrosinase-related protein 2, PCNA proliferating cell nuclear factor, S sense, A; antisense.

### Subcutaneously administration of lupeol to canine melanoma-bearing mice

C.B-17/Icr-scid/scid Jc severe combined immunodeficiency (SCID) mouse were obtained from Crea Japan, INC.. A total of 1×10^7^ cMEL-2 cells were subcutaneously injected into the back regions of SCID mice. Mice whose tumors grew to 10 mm in size were used in this study (day 0). Fifteen mice were randomized into 3 groups on day 0 (n = 5/group). Tumors were removed from 5 mice on day 0 and their weights were measured (day 0 group). Single injections of olive oil (0.1 ml, vehicle control group) or 0.5 mg lupeol in 0.1 ml olive oil (lupeol group) were subcutaneously administered around tumor tissue of each mouse at day 0. The tumor tissues of each mouse were removed 10 days after the injections, and their weights were measured. The experimental plan of the study was approved by the Ethics Committee for Animal Experimentation of Azabu University.

### Statistical analysis

**Figure 1:**
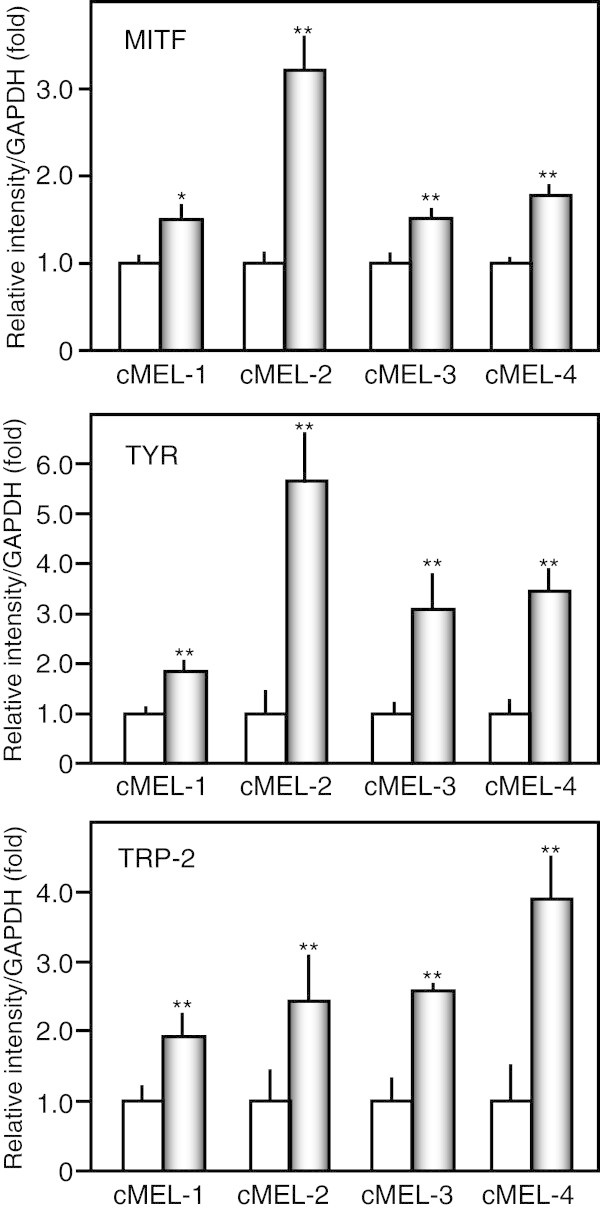
**Up-regulation of melanoma cell differentiation markers by lupeol.** Four canine melanoma cells (2 × 10^5^ cells) were treated without (white bar) or with 5 μM lupeol (gray bar) for 48 h, and the gene expression of MITF, TYR, and TRP-2 was measured by real-time RT-PCR. * *p* < 0.05, ** *p* < 0.01 vs untreated cells (n = 3).

**Figure 2:**
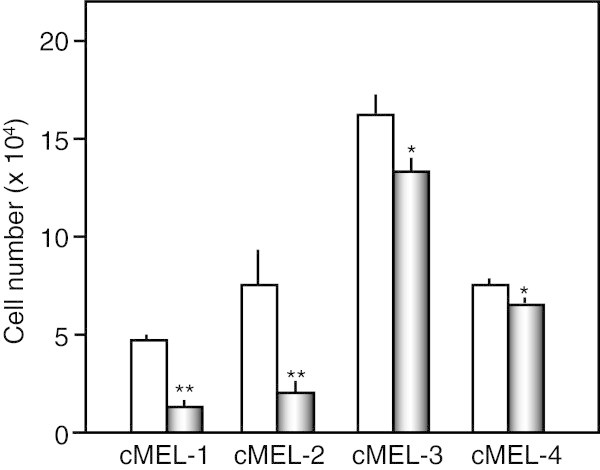
**Antiproliferative activity against 4 canine melanoma cells.** Canine melanoma cells (1 × 10^4^ cells) were treated without (white bar) or with 10 μM lupeol (gray bar) for 4 days, and the viable cell number was subsequently counted by the Trypan blue exclusion method. * *p* < 0.05, ** *p* < 0.01 vs untreated cells (n = 3).

**Figure 3:**
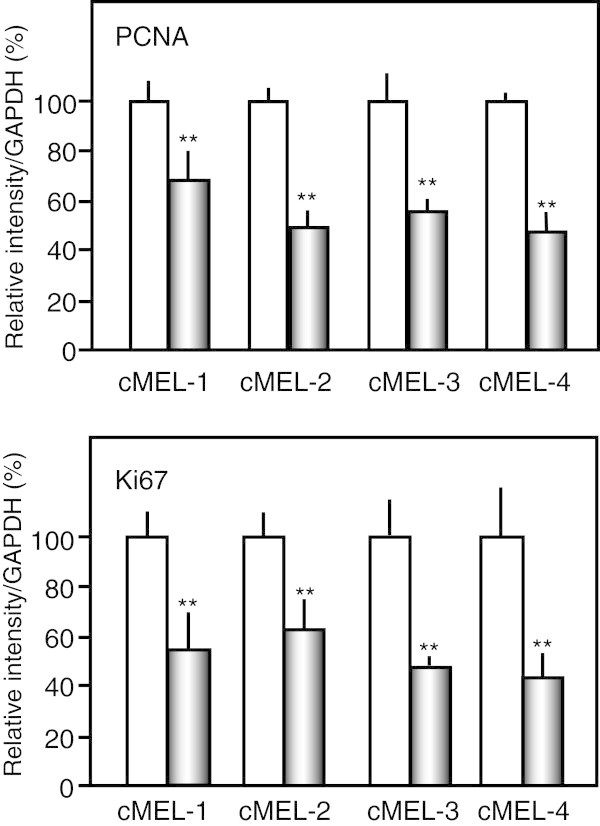
**Attenuation of the expression of PCNA and Ki67 genes by lupeol.** Four canine melanoma cells (2 × 10^5^ cells) were treated without (white bar) or with lupeol at IC_50_ values against each melanoma cell (gray bar) for 48 h, and the gene expression of PCNA and Ki67 was measured by real-time RT-PCR. ** *p* < 0.01 vs untreated cells (n = 3).

**Figure 4:**
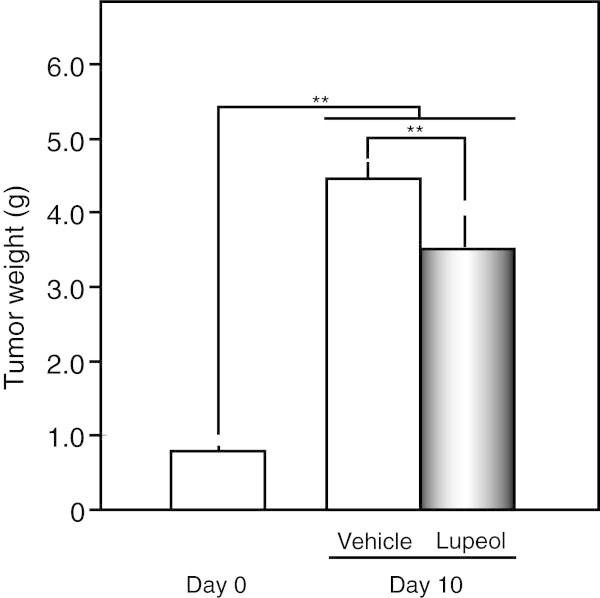
**Suppressive effects of the systemic administration of lupeol on tumor growth in melanoma-bearing SCID mice.** Single injections of olive oil (vehicle control group; Vehicle) or lupeol (lupeol group; Lupeol) were subcutaneously administered to each mouse on day 0. The weights of tumor tissues were measured 10 days after the injections. ** *p* < 0.01 between each group (n = 5).

Data are expressed as the mean ± standard deviation (SD). The significance of differences was analyzed using the Student’s t-test (Figures 1, 2, 3) and one-way ANOVA with Tukey’s multiple comparison test (Figure 4). A value of *p* < 0.05 was considered significant.

## Results and discussion

We investigated whether lupeol induced the differentiation of canine oral malignant melanoma (cMEL) cells by evaluating some differentiation markers with real-time RT-PCR. Figure 1 shows the effects of 5 μM lupeol on the gene expression of microphthalmia-associated transcription factor (MITF), tyrosinase (TYR), and tyrosinase-related protein-2 (TRP-2) in 4 canine melanoma cells. Lupeol up-regulated MITF genes 1.5-3.1-fold, TYR genes 1.8-5.7-fold, and TRP-2 genes 1.9-3.9-fold, respectively, which indicated that the agent induced the differentiation of canine melanoma cells as well as mouse melanoma cells.

The proliferation of 4 canine melanoma cells was markedly suppressed by 10 μM lupeol, and this agent exhibited particularly strong anti-proliferative effects on cMEL-1 and -2 cells (Figure 2). The IC_50_ values of lupeol against 4 melanoma cells were 5.6 μM (cMEL-1), 2.3 μM (cMEL-2), 15.7 μM (cMEL-3), and 15.7 μM (cMEL-4), respectively; however, even at each IC_50_, all melanoma cells did not detach and remained >95% viable, suggesting that the inhibition of cell growth by lupeol can be attributed to its cytostatic (differentiation-inducing), but not cytotoxic effects.

PCNA is a cell-cycle regulator expressed in the nucleus of proliferating cells. The degree of PCNA expression has been correlated with tumor progression and grade in some tumors (Zeng and Davis 2003; Hall et al. 1990; Malham et al. 2010). Ki67 is also a cell proliferation marker, and the expression of Ki67 in the numerous melanoma tissues has been correlated with the presence of malignancy and prognosis (Gould Rothberg et al. 2009). We previously reported that lupeol suppressed the expression of PCNA and Ki67 in B16 mouse melanoma cells both *in vitro* and *vivo*. Therefore, we studied the effects of lupeol at each IC_50_ value against canine melanoma cell growth on the expression of PCNA and Ki67 genes. Real-time RT-PCR analysis revealed that lupeol suppressed the expression of PCNA and Ki67 in 4 canine melanoma cells by 47.4-68.0% and 43.4-62.5%, respectively (Figure 3).

Furthermore, we transplanted cMEL-2 cells into a SCID mouse, and studied the anti-progressive effects of lupeol on tumor tissue (Figure 4). Although the melanoma was 5.7-fold bigger by day 10, growth was significantly suppressed by 78.7% that of the vehicle control with the single administration of lupeol (*p* < 0.01). We administered lupeol to melanoma-bearing mice only once in the present study, and a higher dose and/or repeated injection of lupeol appeared to be more effective in treating the melanoma.

We demonstrated the differentiation-inducing and anti-proliferative activities of lupeol on 4 canine melanoma cells both *in vitro* and *in vivo*. We intend to establish a nonsurgical treatment for canine melanoma with lupeol based on these results.
